# Parasitological efficacy of seasonal malaria chemoprevention in Nampula, northern Mozambique

**DOI:** 10.1093/trstmh/traf127

**Published:** 2025-11-13

**Authors:** Craig Bonnington, Mercia Sitoe, Ivan A Pulido Tarquino, Sonia M Enosse, Chayanin Sararat, Kanokorn Suwannasin, Stephane Proux, Urairat Koesukwiwat, Joel Tarning, Mallika Imwong, Katherine Theiss-Nyland, François Henri Nosten, Nicholas John White

**Affiliations:** Centre for Tropical Medicine and Global Health, Nuffield Department of Medicine, University of Oxford, OX3 7LJ, UK; Malaria Consortium, Avenida Elias Lucas Kumato, 118 - Maputo, Mozambique; Malaria Consortium, Avenida Elias Lucas Kumato, 118 - Maputo, Mozambique; Malaria Consortium, Avenida Elias Lucas Kumato, 118 - Maputo, Mozambique; Mahidol‐Oxford Tropical Medicine Research Unit, Faculty of Tropical Medicine, Mahidol University, Bangkok 10400, Thailand; Department of Molecular Tropical Medicine and Genetics, Faculty of Tropical Medicine, Mahidol University, Bangkok 10400, Thailand; Shoklo Malaria Research Unit, Mahidol‐Oxford Tropical Medicine Research Unit, Mae Sot, Thailand; Mahidol‐Oxford Tropical Medicine Research Unit, Faculty of Tropical Medicine, Mahidol University, Bangkok 10400, Thailand; Centre for Tropical Medicine and Global Health, Nuffield Department of Medicine, University of Oxford, OX3 7LJ, UK; Mahidol‐Oxford Tropical Medicine Research Unit, Faculty of Tropical Medicine, Mahidol University, Bangkok 10400, Thailand; Department of Molecular Tropical Medicine and Genetics, Faculty of Tropical Medicine, Mahidol University, Bangkok 10400, Thailand; Malaria Consortium, Avenida Elias Lucas Kumato, 118 - Maputo, Mozambique; Centre for Tropical Medicine and Global Health, Nuffield Department of Medicine, University of Oxford, OX3 7LJ, UK; Mahidol‐Oxford Tropical Medicine Research Unit, Faculty of Tropical Medicine, Mahidol University, Bangkok 10400, Thailand; Shoklo Malaria Research Unit, Mahidol‐Oxford Tropical Medicine Research Unit, Mae Sot, Thailand; Centre for Tropical Medicine and Global Health, Nuffield Department of Medicine, University of Oxford, OX3 7LJ, UK; Mahidol‐Oxford Tropical Medicine Research Unit, Faculty of Tropical Medicine, Mahidol University, Bangkok 10400, Thailand

## Abstract

**Background:**

Deployment of seasonal malaria chemoprevention (SMC) for young children using monthly sulphadoxine-pyrimethamine-amodiaquine (SPAQ) has recently been extended to Central and East Africa.

**Methods:**

A pilot pharmacometric assessment was nested within a larger deployment of SMC in a high malaria transmission area in northern Mozambique. SPAQ was given to 460 healthy children in two large villages. Simultaneous filter-paper blood spot malaria quantitative PCRs, blood slide microscopy and antimalarial drug measurements were taken before, then 7 and 28 d after first SPAQ administration.

**Results:**

After SPAQ, parasitaemia prevalence decreased from 68% to 41%. Among children followed successfully for 28 d, malaria parasitaemia prevalence declined from 71% to 44%. Preventive efficacy was 97% for *Plasmodium ovale* and 42% for *Plasmodium falciparum*. Reinfections (N=50 with sufficient DNA for genotyping) and recrudescences (N=3) often grew through high concentrations of desethylamodiaquine, yet all 250 *P. falciparum* isolates genotyped were *Pfcrt* 76K, a molecular marker of 4-aminoquinoline susceptibility. One-third (21/64) of microscopy-detectable breakthrough *P. falciparum* infections had patent gametocytaemia. There was a clear chemoprevention exposure–response relationship evident for desethylamodiaquine, but not for sulphadoxine or pyrimethamine.

**Conclusions:**

In Nampula, northern Mozambique, amodiaquine had low parasitological efficacy and sulphadoxine and pyrimethamine did not contribute significantly to chemoprevention.

## Introduction

Seasonal malaria chemoprevention (SMC) is the administration to young children of treatment doses of antimalarial drugs monthly in areas of highly seasonal intense falciparum malaria transmission to prevent malaria illness. SMC has been deployed successfully across the Sahel region of Africa, a belt of intense seasonal falciparum malaria transmission. SMC has employed mainly the administration of amodiaquine (given over 3 d) and single dose sulphadoxine-pyrimethamine (SPAQ) to children aged 3–59 mo for 3–5 mo each year.^1–6^ These antimalarials have fallen to resistance in many areas but are still considered efficacious in West Africa. With reported elimination half-lives of approximately 3 (pyrimethamine), 7 (sulphadoxine) and 10 d (desethyl amodiaquine), SPAQ is expected to provide effective antimalarial chemoprevention for about 1 mo.^6,7^ Recently, SPAQ SMC deployment was extended across eastern and southern Africa, where falciparum malaria is more drug resistant and less seasonal, following substantial broadening of the WHO recommendations in 2022.^7^ The WHO also removed the previous restrictions on the number of cycles or age. The new guidance was not preceded by regional assessments of chemopreventive efficacy. To assess the parasitological efficacy of SPAQ SMC in northern Mozambique, a pilot pharmacometric antimalarial resistance monitoring (PARM) evaluation was conducted. PARM involves simultaneous measurement of parasite densities (by quantitative PCR [qPCR]) and drug levels in recurrent falciparum malaria.^8,9^

## Methods

### Site selection

This observational study took place in January and February 2022 in two districts, Lalaua and Muecate, in Nampula Province, northern Mozambique. Both are areas of high malaria transmission.^10,11^ The PARM evaluation, based on samples taken on days 0, 7 and 28, was conducted within the WHO chemoprevention efficacy protocol.^12^ This was a pilot pharmacometric evaluation nested within a larger deployment programme. Initially, Lalaua was selected, but a high drop-out rate, access difficulties and increased seasonal migration (compromising the necessary community engagement) meant that implementation quality and supervision for sample collection became challenging. Muecate was then selected as it had similar epidemiological characteristics, was more accessible and had fewer obstacles to SMC implementation.

### Screening and enrolment

Recruitment was community based with follow-up at the health facility. Enrolment was conducted according to the Sampford method,^13^ followed by a simple random selection. Communities within selected health facility catchment areas were randomly selected, and 15 children (one per household) per community from randomly selected households were selected using the bottle method (in the absence of resident lists).

### Inclusion and exclusion criteria

Children aged 3–59 mo were recruited if they were afebrile and well, their parent or carer gave fully informed written consent and was willing and able to comply with the study protocol and blood sampling. Children were excluded if they were unwell, had symptoms of malaria (tympanic temperature ≥37.5°C or a history of fever within 48 h), known allergy to the SMC drugs, had received recently any sulpha-based medication, had received other antimalarial drugs or antibiotics, had severe malnutrition, were HIV positive or were included in any other studies. Children with acute malaria were treated with artemether-lumefantrine.

### Procedures

Upon enrolment, axillary temperature, weight and mid upper arm circumference (MUAC) were measured (day 0), capillary blood samples were taken onto Whatman 31 ET Chromatography (Cat. No. 3031–915) filter papers and standard malaria blood slides were prepared. Observed daily doses of oral amodiaquine (75 mg, infants aged 3–12 mo; 150 mg, children aged 1–5 y) were given for 3 d, and one dose of sulphadoxine-pyrimethamine (12.5 mg pyrimethamine/250 mg sulphadoxine for infants and 25/500 mg for older children) was given on the first day. No additional interventions, monitoring or support were provided. For the PARM evaluation, dry blood spot capillary blood samples on days 7 and 28 were analysed. The filter papers and microscope slides were barcoded, stored under dry conditions and later airfreighted to the analytical laboratories in Thailand.

### Assays

Blood slides were examined by experienced microscopists (Shoklo Malaria Research Unit). The dried filter paper blood spots were used to assess the species of malaria parasites and estimate their density using qPCR.^14,15^ If there was sufficient DNA, molecular genotyping of known drug resistance markers (*Pfcrt, Pfmdr, PfAAT, Pfdhfr, Pfdhps*) was conducted, and paired acute and recurrent isolates were compared using polymorphic alleles in *Pfmsp1, Pfmsp2, glurp* and the microsatellite Poly A.^14^ Identity of one or more alleles at all four loci was regarded as a definite recrudescence, and identity at three of four or two of three successfully amplified alleles was regarded as a possible recrudescence. Exact areas of filter paper dry blood spots were punched automatically and then extracted and assayed to measure drug levels (amodiaquine [AQ], desethyl amodiaquine [DAQ], pyrimethamine [PYM] and sulphadoxine [SDX]) using validated liquid chromatography with tandem mass spectrometry assays.^16^ The lower limits of quantitation were sulphadoxine 840 ng/mL, pyrimethamine 4.42 ng/mL, amodiaquine 1.87 ng/mL and desethylamodiaquine 2.95 ng/mL.

### Statistical analysis

SMC efficacy over 28 d was assessed by comparing qPCR malaria prevalence before and after treatment. Antimalarial blood concentration profiles between D7 and D28 were estimated using a Bayesian hierarchical linear model, incorporating inter-individual variability in both the intercept and slope and accounting for left censoring of values below the lower limit of quantitation (LLOQ). Drug elimination half-life was derived from t_1/2_=log(2)/decay rate.

## Results

### Characteristics of participants

**Figure 1.**
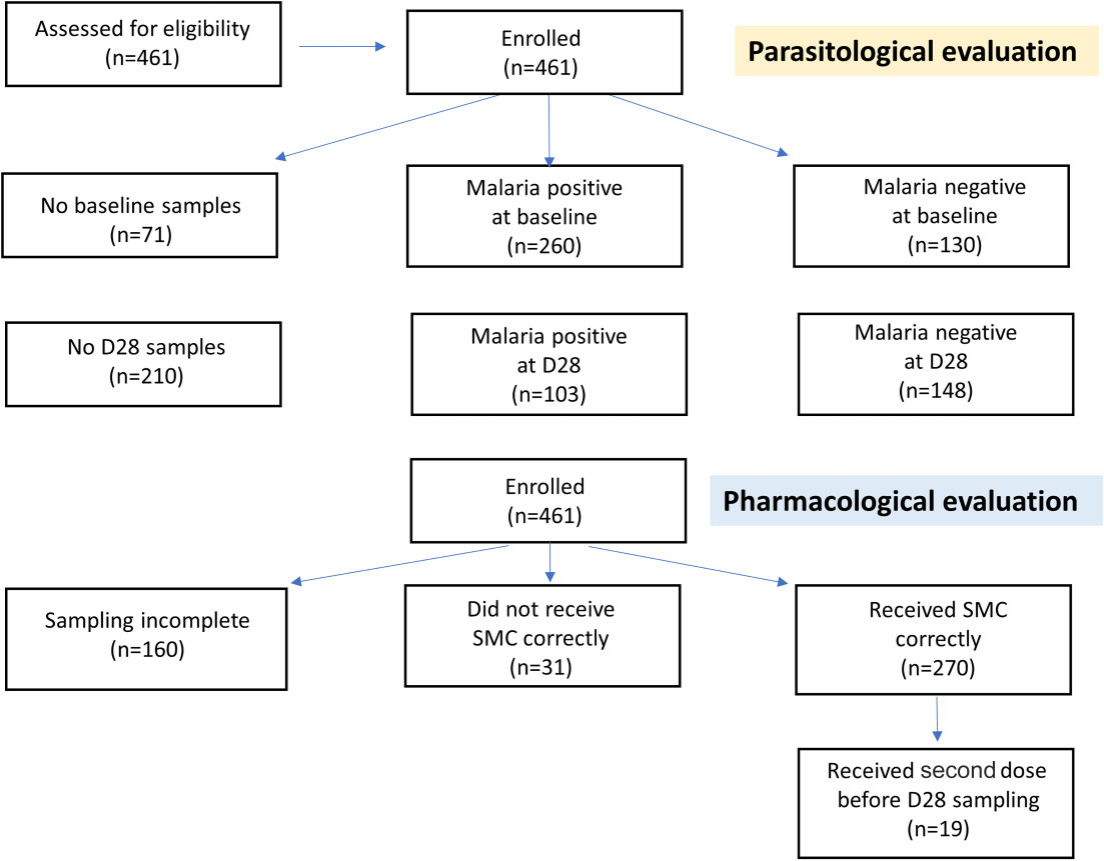
Diagram of participant flow in the trial. Malaria positive refers to malaria parasitaemia. SMC: seasonal malaria chemoprevention.

Of 461 studied children, 183 were from Lalaua and 278 from Muecate (Figure 1, Table 1). Ages ranged from 3 to 59 (mean 32, median 33) mo and were similar in the two sites. The median (IQR) z-scores for weight and MUAC were −0.8 (−2.3 to 0.7) and −0.3 (−1.1 to 0.4), respectively (Figure S1). In general SMC was well tolerated, with no serious adverse events or hospitalisations. Six children vomited their medication but it was readministered successfully. Unfortunately, the reporting and treatment of intercurrent febrile illnesses was not documented systematically.

**Table 1.** Demographic characteristics of participants

	District		
	Lalaua	Muecate	Overall
Number	183	278	461
Age (mo)	30.3 (14.4)	32.6 (15.0)	31.7 (14.8)
Male	78 (43%)	132 (47%)	210 (46%)
Female	105 (57%)	146 (53%)	251 (54%)
Weight (kg)	10.7 (9.3–13.3)	13.0 (9.9–15.7)	12.7 (9.8–15.2)
Z-score weight	−1.4 (−2.4 to −0.2)	−0.6 (−2.2 to 0.9)	−0.8 (−2.3 to 0.7)
MUAC (cm)	15 (14–17)	15 (14–16)	15 (14–16)
Z-score MUAC	−0.2 (−0.9 to 0.5)	−0.3 (−1.1 to 0.4)	−0.3 (−1.1 to 0.4)

MUAC: mid-upper arm circumference.

Data are presented as n (%), median (IQR) or mean (SD).

### Baseline malaria parasitaemia

**Figure 2.**
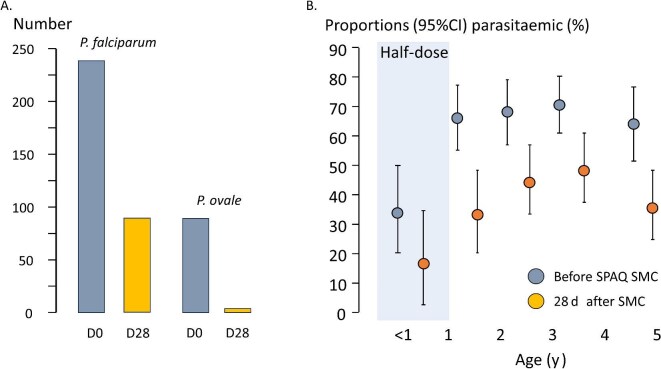
(A) Number of children who were parasitaemic by qPCR before and after SPAQ SMC. In addition, there were two children with *P. vivax* and one with *P. malariae* at D0, but none at D28. Parasites were speciated only when sufficient DNA was available. (B) Stratification of malaria parasitaemia proportions by age, before and after SPAQ SMC. Half-dose refers to the SPAQ dosing for children aged <1 y. Error bars represent 95% CIs. qPCR: quantitative PCR; SMC: seasonal malaria chemoprevention; SPAQ: sulphadoxine-pyrimethamine-amodiaquine.

Of 390 baseline samples (71 children did not have samples taken) from both sites, 260 (67%) were malaria positive by PCR; 241 (62% overall) contained *Plasmodium falciparum* (157 *P. falciparum* mono-infections and 77 mixed) (Figure 1, Tables 2 and 3). Assuming the seven unspeciated samples contained *P. falciparum*, the *P. falciparum* infection prevalence was 64%. Concomitant *Plasmodium ovale* infection occurred in 76 children and there were 12 *P. ovale* mono-infections. Thus, one-quarter of parasitaemic children (88/378, 23% [95% CI 19 to 28%]) had *P. ovale* infections, commonly in combination with *P. falciparum*. There were no *Plasmodium vivax* or *Plasmodium malariae* mono-infections. Older children had a higher prevalence of malaria (Figures 2 and S2). The risk was higher in Muecate than in Lalaua (RR=1.58, p<0.01). Among the parasitaemic children, age, weight and MUAC did not impact parasite density significantly (Figures S2c and S2d).

**Table 2.** Malaria prevalence before and after SPAQ SMC

	Before SMC	28 d after SMC
-		
**PCR positive**	260/390 (67%)	103/251 (41%)
- Lalaua	90/178 (50%)	27/67 (40%)
- Muecate	170/212 (80%)	76/184 (41%)
**Microscopy positive**	165/336 (49%)	54/100 (54%)
- Lalaua	68/168 (40%)	20/27 (74%)
- Muecate	97/168 (58%)	34/73 (47%)
Parasite density/uL* (geometric mean: range)	949 0–105 591	142 0–215 294

qPCR: quantitative PCR; SMC: seasonal malaria chemoprevention; SPAQ: sulphadoxine-pyrimethamine-amodiaquine.

*estimated from qPCR.

**Table 3.** Distributions of malaria species in parasitaemic children before and after SMC

Malaria species	Day 0	Day 28
*P. falciparum*	163 (62.7%)	89 (86.4%)
*P. falciparum + P. malariae*	1 (0.4%)	-
*P. falciparum + P. ovale*	25 (9.6%)	2 (1.9%)
*P. falciparum + P. ovale curtisi*	30 (11.5%)	-
*P. falciparum + P. ovale curtisi + P. ovale wallikeri*	3 (1.2%)	-
*P. falciparum + P. ovale wallikeri*	17 (6.5%)	-
*P. falciparum + P. vivax + P. ovale curtisi + P. ovale wallikeri*	1 (0.4%)	-
*P. falciparum + P. vivax + P. ovale wallikeri*	1 (0.4%)	
P. ovale*	1 (0.4%)	1 (1.0%)
*P. ovale curtisi*	8 (3.1%)	-
*P. ovale wallikeri*	3 (1.2%)	-
Undetermined*	7 (2.7%)	11 (10.7%)
Total	260	103

SMC: seasonal malaria chemoprevention.

*If there was insufficient DNA then the malaria species could not be determined.

Of the 336 patients with baseline blood slides, 139 could not be read satisfactorily, 28 were negative and 159 were positive for *P. falciparum* (58 with both trophozoites and gametocytes). Thus, the majority (90%) of children who were PCR positive at enrolment were also microscopy positive (i.e. parasite density >50/µl).

### Parasitological findings following SMC: day 28 comparison

Of 251 children with D28 samples, 103 (41%) were positive for malaria parasites (Figure 2, Table 2); absolute reduction 26% and relative reduction 39% in parasitaemia prevalence. In a paired comparison of samples available for both D0 and D28, parasitaemia prevalence before was 71.2% (141/198) vs 44.4% (88/198) after SMC; RR 0.62 (95% CI 0.51 to 0.74) (Table S1). There were no obvious differences in age or baseline parasite densities between children with and without D28 samples. Of the 92 speciated recurrences, 91 were *P. falciparum* (89 as mono-infections) and three were *P. ovale* (two mixed, one mono-infection) (Table 3). The reduction in *P. ovale* prevalence (RR 0.05; 95% CI 0.01 to 0.14) was substantially greater than for *P. falciparum* (RR 0.59; 95% CI 0.49 to 0.70); ratio of risk ratios 0.08. The prevalence of malaria parasitaemia declined more in Muecate (80% [170/212] before SMC, 41% [76/184] at D28 [Table 2]) than it did in Lalaua (50% [90/178] before SMC, 40% [27/67] at D28). Also, 78 children remained malaria positive despite SMC; 50 were identified as new infections, three were recrudescences based on a ‘strict classification’ (identical at each allele) (Table S2) and 25 recurrence isolates could not be genotyped satisfactorily because of insufficient DNA. Using a less strict classification (three of four alleles similar) there were 28 possible recrudescences.

There was good correlation between baseline PCR and microscopy density estimates (Figure S3), so only the PCR-positive slides were read at D28. Of the 100 available D28 slides, 54 were positive by microscopy (geometric mean: 303 per µl: range: 16 to 122 080 per µl), 24 tested negative and 22 were unreadable. In the microscopy-positive children, 23 (43%) were gametocytaemic, 40 had asexual-stage parasites, nine had both gametocytes and asexual-stage parasites and 14 had only gametocytes.

### Antimalarial drug concentrations following SMC

**Figure 3.**
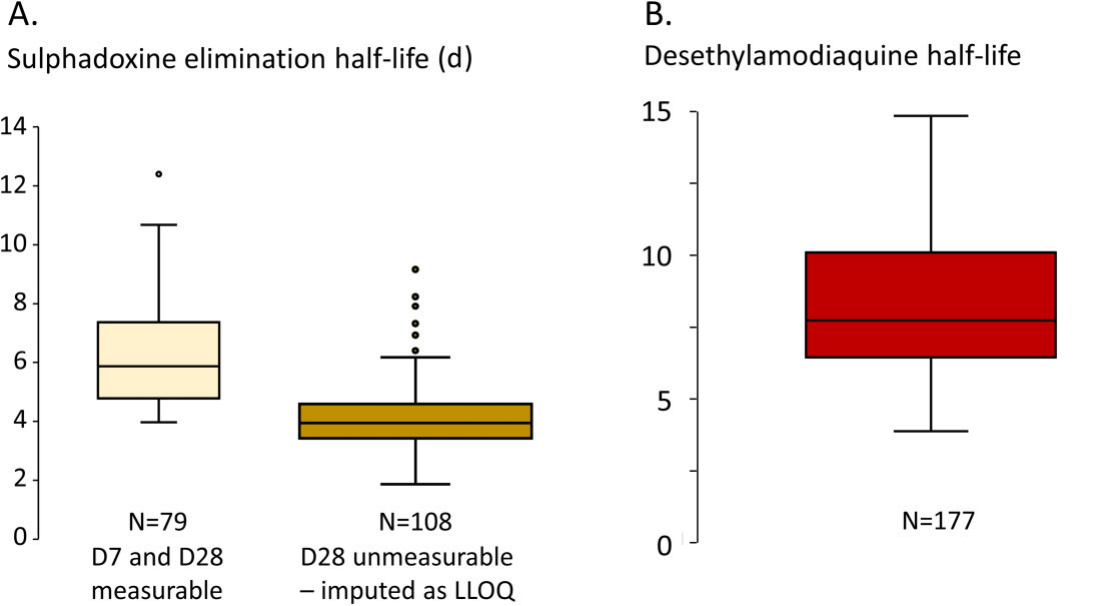
Elimination half-life estimates derived from the D7 and D28 concentrations for (A) sulphadoxine and (B) desethylamodiaquine. As many sulphadoxine D28 measurements were below the lower limit of quantitation (LLOQ), the estimates were made only on those paired samples that were both measureable (A, left) or by imputing the D28 level as the LLOQ threshold (A, right). Median, IQR and 95th centiles shown.

The drug measurements identified operational issues at the Lalaua site with 28/102 (27%) children having no detectable D7 sulphadoxine and pyrimethamine levels (Figures 1 and S4). Most (24 of 28) of these children also had undetectable D7 desethylamodiaquine levels (Figure S4), indicating that they had not received SMC. By comparison, SMC administration at Muecate was good; only 3/199 (2%) children at Muecate had unmeasurable D7 sulphadoxine and pyrimethamine levels. The geometric mean capillary blood D7 sulphadoxine level was 19 402 (range 1300 to 76 100) ng/mL. Approximately one-half of the D28 levels were below the limit of accurate quantitation (106/248), as expected (Figure S4, Table S3). Pyrimethamine is more rapidly eliminated so only D7 levels are usually detectable (Figure S4). Amodiaquine is metabolised rapidly into its slowly eliminated bioactive desethyl metabolite, which contributes the majority of the chemosuppressive antimalarial effect. On day 28, amodiaquine was detected in 19 children (geometric mean 14.1 [range: 2.1 to 374] ng/mL), suggesting that these children may have received their second SMC round before the D28 blood sampling. The geometric mean (range) D28 blood desethylamodiaquine concentration was 16.2 (3 to 357) ng/mL. This was slightly lower (12.6 ng/mL) in subjects who were parasitaemic on D28. Fifteen subjects had higher amodiaquine than desethylamodiaquine levels on days 7 and 28 (Figure S4), indicating either mislabelling or drug readministration. After adjusting for measured but left-censored values under the LLOQ, the estimated mean sulphadoxine terminal elimination half-life (t_1/2_β) was 4.1 (95% CI 3.8 to 4.4) d (Figure 3A), and the desethylamodiaquine t_1/2_β was 8 (95% CI 7.6 to 8.5) d (Figure 3B). These values were not correlated (Figure S5).

### Recurrent parasitaemia following SPAQ SMC administration

**Figure 4.**
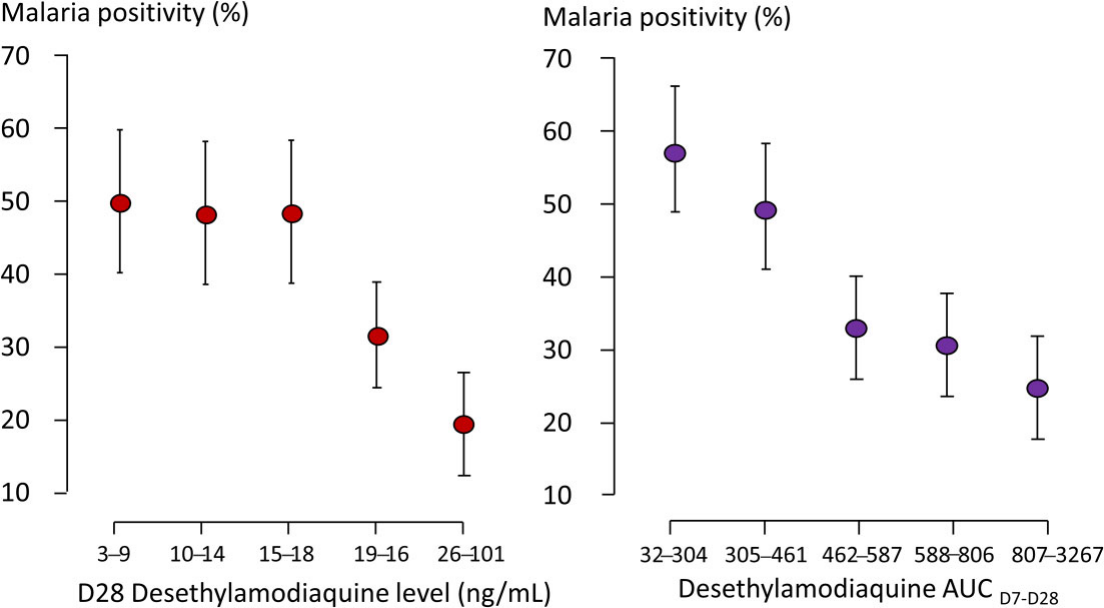
Relationship between (left) D28 desethylamodiaquine blood concentrations and (right) desethylamodiaquine exposure between day 7 and day 28 (AUC_D7-D28_) and the proportion of children in the study with detectable malaria parasitaemia on day 28. Data are represented by proportions and 95% CIs and divided into quintiles.

**Figure 5.**
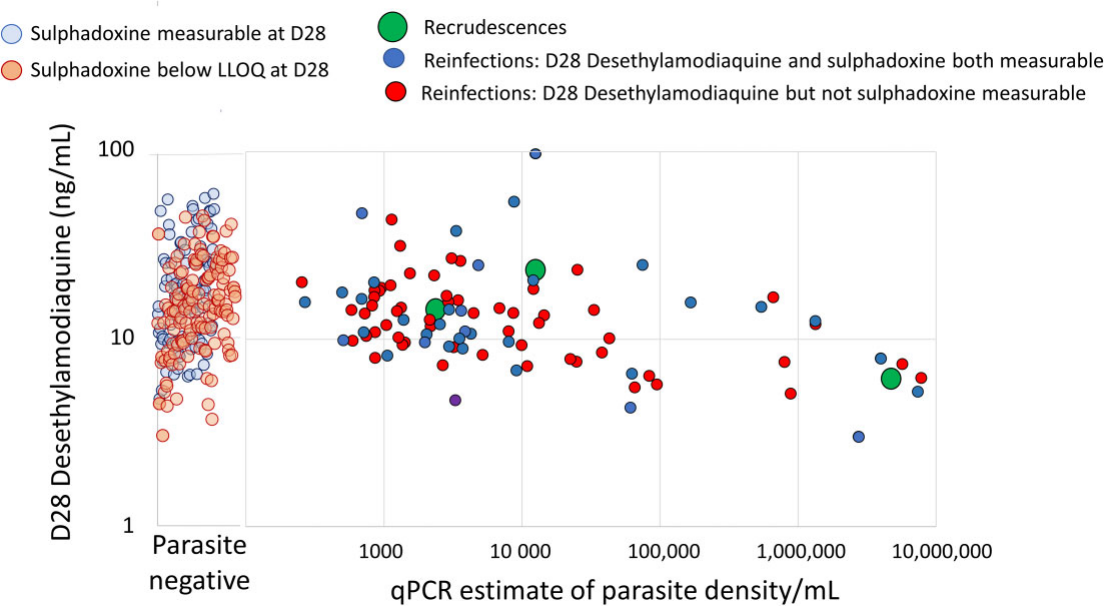
Relationship between D28 parasite density and D28 blood desethylamodiaquine concentrations. Individual results shown. LLOQ: lower limit of quantitation; qPCR: quantitative PCR.

The absence of measurable amodiaquine and pyrimethamine, and the presence of desethylamodiaquine and often sulphadoxine in the D28 sample, are consistent with the correct implementation of SMC. Measurements suggesting incorrect drug administration were excluded for the analysis of drug levels. Malaria prevalence and parasite densities were correlated with previous and concomitant drug levels (Figures 4 and 5 and Figures S6 to S9). Finding malaria parasites and antimalarial drugs in the same blood sample indicates that the parasites could grow in that drug concentration (i.e. the measured blood concentration was below the in vivo minimum inhibitory concentration [MIC]). PCR-detected D28 parasitaemia, despite detectable desethylamodiaquine levels in the concomitant sample (median 13.6, IQR 9.41 to 18.4 ng/mL), was documented in 70 subjects. In 12 (16%) cases the D28 parasitaemia was higher than before SMC. The three definite asymptomatic parasitaemia recrudescences had similar D28 desethylamodiaquine and sulphadoxine concentrations to the other subjects (i.e. they were genuine treatment failures) (Figure 5, Table S2). The D28 parasite samples of 25 children could not be genotyped as there was insufficient DNA, or the paired sample was unavailable.

Higher D28 desethylamodiaquine levels were associated with lower malaria prevalence (Figure 4, Figures S6a and S6b), but there was no clear relationship for sulphadoxine, pyrimethamine or amodiaquine (Figures 5 and S7-S9). Nevertheless, parasite densities determined by microscopy as high as 1% occurred despite adequate desethylamodiaquine exposures (the highest was 56 160/µl with a concomitant D28 desethylamodiaquine level of 35.4 ng/mL) (Figure 5). The prevalence of D28 parasitaemia was lower in infants (aged <12 mo) than it was in older children (Figure 2) and only two of the 17 infants who had weight and drug levels measured were parasite positive on D28. In the older children (i.e. aged 1–5 y) who received the full SPAQ dose there was a weak relationship between body weight and D28 parasitaemia prevalence (Figure S10), but a stronger relationship with desethylamodiaquine exposure (Figure 4).

### Determinants of breakthrough malaria following SMC

Sulphadoxine (D7 and D28), pyrimethamine (D7) and amodiaquine (D7) levels were not associated significantly with recurrent parasitaemia, although their levels were correlated with desethylamodiaquine levels. D28 malaria positivity was associated only with desethylamodiaquine levels (Figure S11). In a logistic model adjusting for age, geographical location and calendar time (Table 4), higher D28 desethylamodiaquine levels were associated with a reduced probability of malaria recurrence (p=0.009) (Figures S6a and S6b). D28 desethylamodiaquine concentrations of 25 ng/mL were associated with a predicted recurrence probability of 46%, but 75% of children had D28 levels <25 ng/mL (Figure 4). Day 7 and the area under the blood concentration time curve (AUC)_7-28_ desethyamodiaquine levels were closely correlated (Figure S12). D28 desethylamodiaquine levels were only weakly correlated with weight, age and MUAC.

**Table 4.** Multiple logistic regression model examining the determinants of PCR-detected D28 parasitaemia

Variable	Coefficient	p
Intercept	−2.7042	0.1552
Age (mo)	−0.0862	0.0045
Weight (kg)	0.5846	0.0019
Z-score of weight	−0.8604	0.0030
AUC from D7 to D28 of desethylamodiaquine	−0.0884	0.0070

AUC: area under the blood concentration time curve.

### Molecular markers of antimalarial drug resistance

All 250 *P. falciparum* isolates evaluated were *Pfcrt*_72-76_ CVNMK (i.e. wild type). None of the downstream *Pfcrt* mutations associated with piperaquine resistance were found. The majority of parasites were *Pfmdr1* wild type at positions 86, 184, 784, 945, 1034, 1042, 1068, 1197, 1246 and 1314. The 184F mutation was found in 25 isolates (10 alone and 15 in mixtures) and three isolates were 1197N. For *Pf*AAT (N=49) seven isolates were mutant (*Pf*AAT 258L) and a further eight isolates contained mutant parasites in mixtures. All were wild type at *Pf*AAT 313.

All 49 isolates tested were *Pfdhfr* wild type at positions 16 and 164 and mutant at *Pfdhfr* 108 (N), 51(I) and 59(R), although four infections were mixed with wild type *dhfr* 59C parasites. All 49 isolates were *Pfdhps* wild type at positions 431, 581 and 613. For *Pfdhps* 436 all were wild type but two contained mixtures with mutant 436F (one) and 436A (one). All but one of the isolates were mutant *Pfdhps* 437G, although nine contained mixtures with *Pfdhps* 437 wild type. All but one isolate contained mutant *Pfdhps* 540E, although 11 also contained wild type *Pfdhps* 540K. Thus, the majority of *P. falciparum* isolates had the ‘quintuple *dhfr/dhps* mutant’ haplotype prevalent throughout much of East Africa.

## Discussion

In the Sahel region of Africa, SMC has been shown in clinical trials to reduce symptomatic malaria and anaemia and provide a moderate reduction in severe malaria.^2–5,17^ As deploying SMC represents a substantial investment of human and financial resources for National Malaria Control programmes it is essential that it is effective. Just as antimalarial treatment requires therapeutic assessment, so does SMC.^8,9,12,18,19^ The WHO recommends that SMC should have a minimum of 75% preventive efficacy at 28 d,^7^ although the definition of preventive efficacy in this recommendation is unclear. SPAQ SMC has recently been deployed in East Africa where drug resistance is much worse than in West Africa. In the first studies from this region, conducted in Uganda,^20^ SPAQ was reportedly highly effective, with efficacy in preventing clinical malaria which surpassed that reported earlier in West Africa.^2–4,17^ This first prospective use of PARM in SMC^8^ shows that SPAQ SMC has poor parasitological chemoprevention efficacy in northern Mozambique. *Plasmodium falciparum* parasites in young children were often able to grow through relatively high drug concentrations and produce mature (presumably transmissible) gametocytes (Figure 5). Approximately 40% of children were parasitaemic 28 d after SMC deployment.

Northern Mozambique is an area of high malaria transmission.^21^ Two-thirds of children had asymptomatic *P. falciparum* and one-third had *P. ovale* infections before SMC. SPAQ chemoprevention parasitological efficacy for *P. falciparum* was low (42%), but for *P. ovale* it was excellent (97%). Immune subjects usually clear malaria parasitaemia promptly, even with relatively ineffective antimalarial treatments,^9,17^ yet in at least three cases *P. falciparum* recrudescences occurred despite adequate drug exposures. The accuracy of genotyping comparisons in areas of high transmission is reduced,^22^ particularly in asymptomatic *P. falciparum* infections where the parasite densities of different infecting ‘clonal populations’ are likely to be similar (unlike in symptomatic infections). Using less stringent criteria for genotype comparisons resulted in a much higher number of estimated *P. falciparum* recrudescences.

Sulphadoxine and desethylamodiaquine (the active metabolite of amodiaquine) were both eliminated rapidly in young children.^23,24^ Based on the relationship between drug concentrations and the presence or absence of parasites in the same sample there was no convincing evidence that either sulphadoxine or pyrimethamine contributed to the suppression of *P. falciparum* parasitaemia. By contrast, there was a clear exposure-related chemopreventive effect of amodiaquine. The D_7-28_ desethylamodiaquine AUC was a better predictor of the chemopreventive response than either the individual D7 or D28 levels. Older children had a lower risk of breakthrough infections, which suggests that increasing immunity compensates for reduced drug exposure in determining therapeutic responses to chemoprevention antimalarial drugs. Lower weight for age and lower arm circumference were both weak independent risk factors for breakthrough infections. Rapid drug elimination probably contributes to the overall low parasitological efficacy of SPAQ against *P. falciparum*.^25,26^

The high level of parasitological *P. falciparum* chemoprevention failure is not explained adequately by the known drug resistance markers. Although multiple mutations in *Pfdhfr* and *Pfdhps* were common (the main haplotype was the widely prevalent ‘quintuple’ triple *dhfr* and double *dhps* mutants^25^), these mutations would be expected to reduce but not abolish the antimalarial effect of the sulphadoxine-pyrimethamine component. Nevertheless, even without SP, amodiaquine alone should have been highly effective in chemoprevention as all genotyped *P. falciparum* parasites were ‘wild type’ at position 76 in the *Pfcrt* gene (haplotype CVIEK), that is, they did not have the key causal mutation (*Pfcrt* 76T) conferring 4-aminoquinoline resistance.^27–30^ Loss of this key ‘chloroquine resistance mutation’ may have reached fixation in parts of Southeast Africa. The failure of desethylamodiaquine to suppress parasite multiplication despite the molecular signature of 4-aminoquinoline susceptibility suggests that there could be other genetic factors contributing to amodiaquine chemoprevention failure. This will clearly need further investigation.

This study has several important limitations and weaknesses. This was a pilot study in one area of northern Mozambique. It is uncertain if the results are generalisable, although the molecular markers of drug resistance in *P. falciparum* are similar to those elsewhere in East Africa. There was selection bias and there were operational constraints. Vomiting of tablets was infrequent but may have been under-reported. The key measure of illness prevention could not be assessed reliably. It is possible that clinical efficacy was superior to parasitological efficacy. Microscopy probably underestimated patent infections because slide quality was poor. Drug level measurements identified significant operational issues at the initial study site. SMC effectiveness was substantially reduced as a consequence. Although this did not affect the overall conclusions regarding therapeutic efficacy, as incorrectly dosed subjects were excluded from the relevant analyses, it reduces confidence significantly in the estimates from that site, and it contributed overall to the poor performance of SMC. Fortunately, the other site performed relatively well, and the unanticipated ‘internal control’ of *P. ovale* infections, for which SPAQ chemoprevention was highly efficacious, provided a robust comparator. The different pharmacokinetic properties of the four drug analytes allowed triangulation and root cause analysis to identify errors in drug administration and study conduct. The most common error was sampling blood just after rather than before drug administration. But some subjects did not receive SMC as they should have, follow-up was incomplete and a substantial proportion of children could not be contacted to provide D28 samples. Overall, this weakens but does not invalidate the conclusions. Even if there are study conduct errors, finding malaria parasites with adequate concentrations of drug in the same blood sample and the excellent efficacy in *P. ovale* prevention provides clear evidence of SPAQ parasitological failure in *P. falciparum* chemoprevention. In about one-third of assessable cases, the breakthrough blood stage infections had been established for long enough to generate detectable and, therefore, presumably transmissible densities of gametocytes. This reflects the drug resistance selection pressure.

The primary objective of chemoprevention is the prevention of illness and death from malaria. It is sometimes argued that antimalarial parasitological efficacy underestimates clinical efficacy in areas of high transmission. On the other hand, spontaneous resolution of malaria is usual in immune individuals and this overestimates the clinical efficacy of failing antimalarial drugs in younger less immune subjects. However, suppressive efficacy does require an antimalarial effect. At the two study sites in northern Mozambique, the antimalarial effect provided by amodiaquine was weak, and there was no pharmacometric evidence that sulphadoxine-pyrimethamine contributed at all. The common finding of microscopy patent gametocytaemic *P. falciparum* infections, which commonly followed SPAQ chemoprevention, must reflect a resistance selection pressure, and parasite densities as high as 1% occurred despite adequate desethylamodiaquine concentrations. With such extensive roll-out of chemoprevention in areas with high pre-existing levels of drug resistance, it is essential that monitoring of antimalarial chemopreventive efficacy is performed. More information on the clinical correlates of the weak SPAQ chemosuppressive efficacy is required urgently.

## Supplementary Material

traf127_Supplemental_File

## Data Availability

Data are available on request to the Malaria Consortium.
