# Supplementary material for: Parasitological efficacy of seasonal malaria chemoprevention in Nampula, northern Mozambique
Source: Trans R Soc Trop Med Hyg. 2025 Nov 13;120(3):258–67. doi: 10.1093/trstmh/traf127 (PMC13017671; doi:10.1093/trstmh/traf127)
Supplement: traf127_Supplemental_File [file traf127_supplemental_file.pdf]

# Supplementary

**Appendix**

*Multiple Regression Models assessing the contributors to breakthrough parasitaemia*

The MLR equation is written as the generalized linear model for the $logit$ function as follows:

$$logit(p) = \beta_0 + \beta_1 x_1 + \beta_2 x_2 \ldots\ldots\ldots + \beta_m x_m$$

where, $p$ is the probability of having positive parasitaemia on D28, $\beta_0$ is the intercept, and $\beta_i$ is the regression coefficient indicating the relative effect of variable $x_i$. The values of model parameters were determined by maximizing the log likelihood function across $n$ observations of outcomes

$$l(p) = \sum_{i=1}^{n} y_i log(p) + (1 - y_i) log(1 - p)$$

where, $y_i = 1$ when D28 parasitaemia was positive and $y_i = 0$ when D28 parasitaemia was negative. The MLR analysis was performed by constructing models using nine possible predictors; age, weight, z-score of weight, MUAC, z-score of MUAC, D7 capillary blood levels of sulphadoxine, pyrimethamine, and desethylamodiaquine, and the AUC from D7 to D28 of desethylamodiaquine levels. A total of 511 models were constructed using all possible combinations of the nine variables. The models were fitted with the same dataset from 162 patients.

## Supplementary Figures

**Figure S1:** Demographic characteristics of participants. (a) Age distribution by district. Solid lines indicate medians and dashed lines represent interquartile ranges (IQRs). (b) Body weights and corresponding z-scores. (c) Mid-upper arm circumferences (MUAC) and corresponding z-scores.

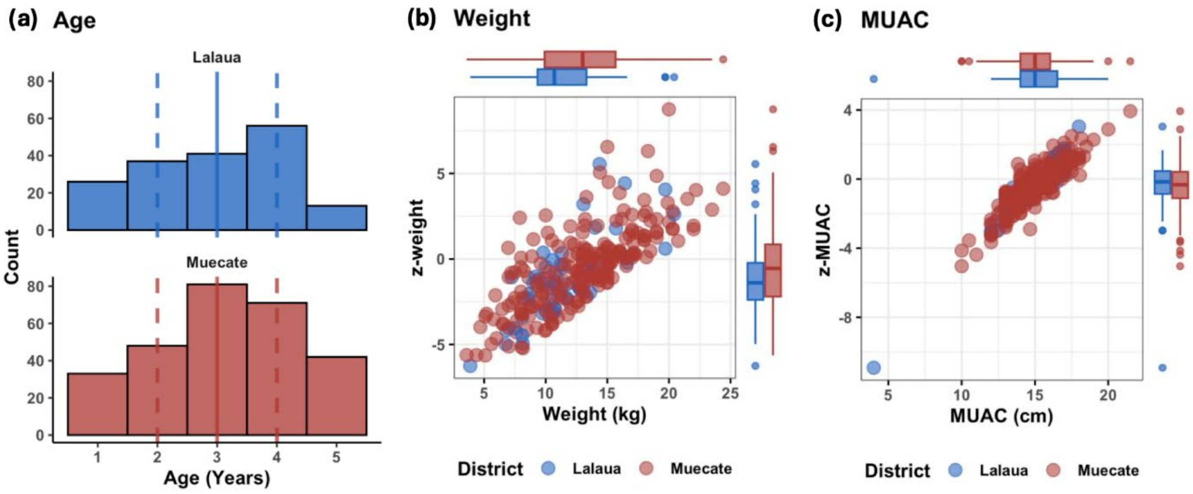

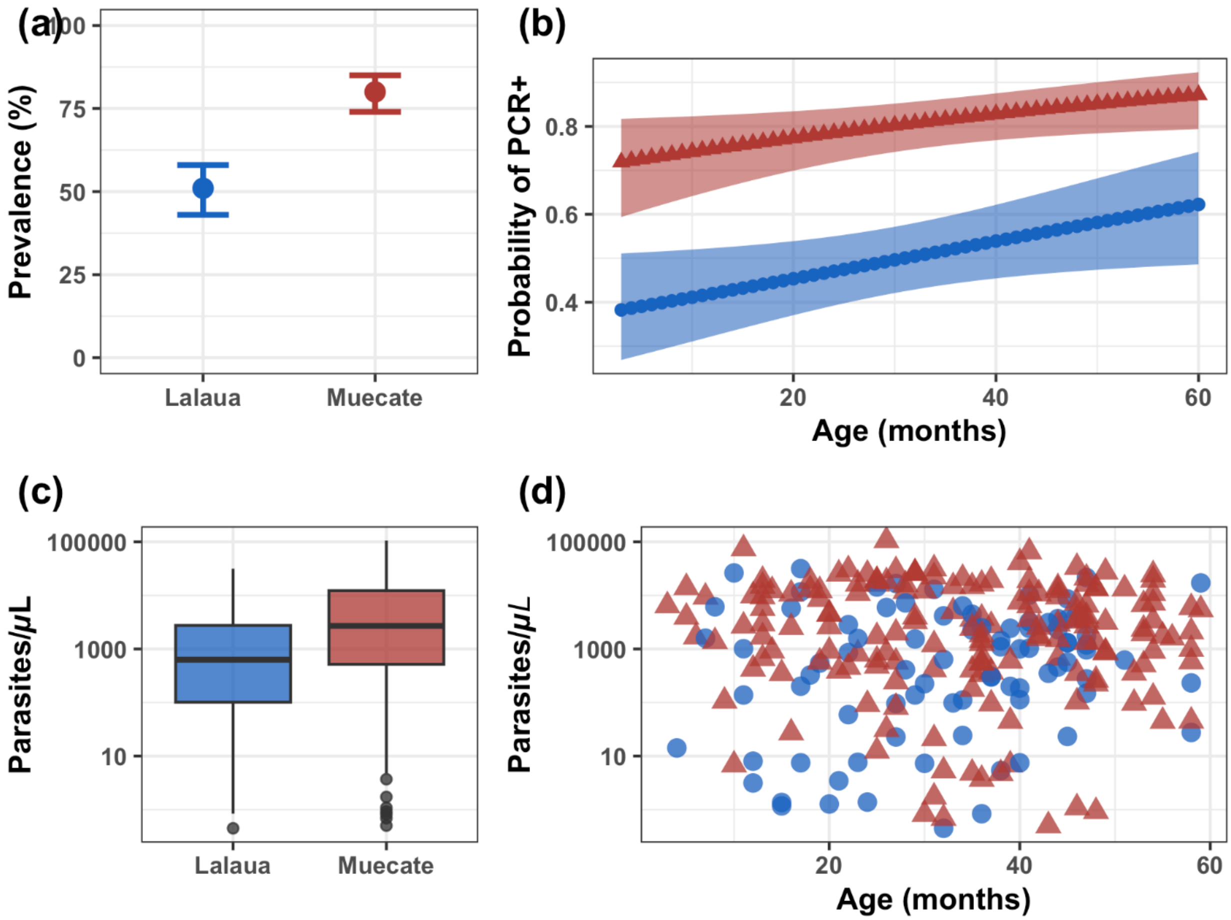


**Figure S2:** Baseline malaria characteristics. **(a)** Malaria prevalence by district, with 95% confidence intervals (CIs). **(b)** Estimated probability of testing PCR-positive on Day 0. **(c)** Geometric mean (95%CI) baseline parasite densities by site. **(d)** Baseline parasite densities stratified by age (blue circles-Lalaua, red triangles Muecate).

**Figure S3**: Correlation between microscopy and qPCR estimates of parasite densities

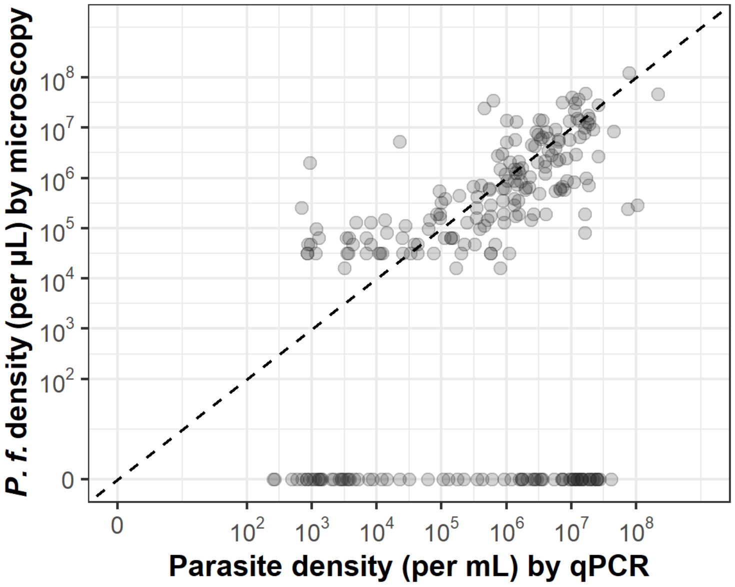

**Figure S4**: Capillary whole blood drug levels by site. Sulphadoxine and pyrimethamine are co-formulated in the same tablet. Desethylamodiaquine is the bioactive desethyl metabolite of amodiaquine.

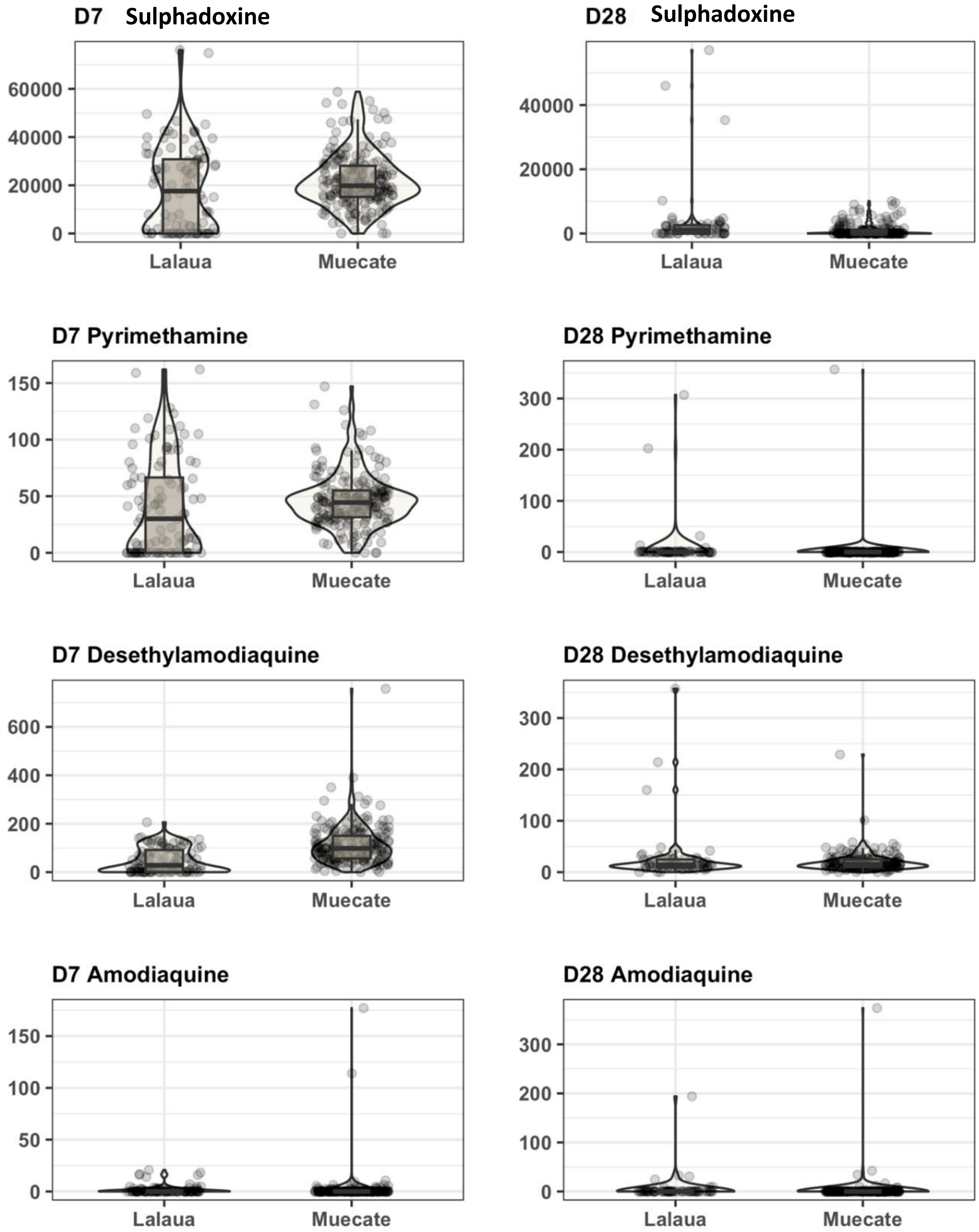

**Figure S5a:** Predicted probability of testing malaria-positive at D28 as a function of (right) D28 desethylamodiaquine concentration and (left) desethylamodiaquine exposure between day 7 and day 28. (Top) Predicted probabilities were estimated using a logistic regression model that adjusts for age (modeled with a smooth term), geographical location, and calendar time. The predictions assume a median child age of 33 months, in Muecate district, on 2022-02-25. Shaded areas represent the 95% confidence interval (CI). **(Bottom)** Distribution of D28 desethylamodiaquine concentrations and desethylamodiaquine exposures. Histogram counts (pale grey) are shown on the right y-axis, while the cumulative count is displayed on the left y-axis (thick line).

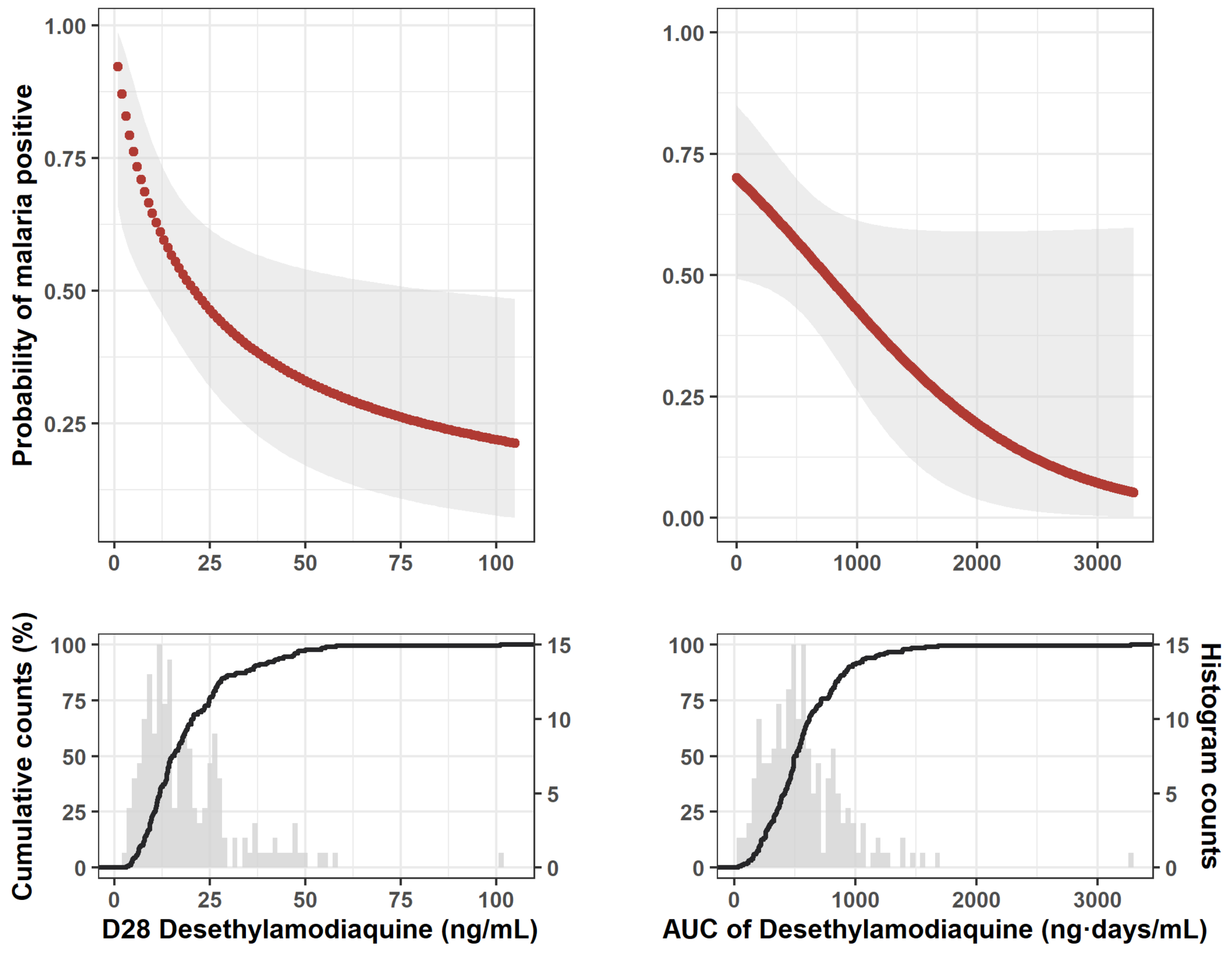

**Figure S5b**. The probability of an average child in the Mozambique study sites (aged 33 months old, weight 12.7 kg, Z-score = -0.81) being parasitaemic on D28 in relation to the $AUC_{7-28}$ of whole blood desethyl amodiaquine (DAQ) levels derived from the multiple regression model. Dashed lines represent 95%CI.

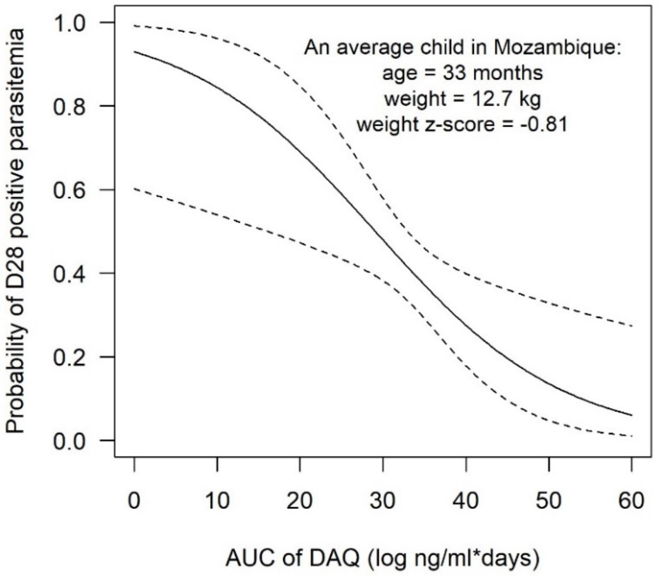

**Figure S6**. Day 7 whole capillary blood sulphadoxine concentrations in children whose blood samples were malaria parasite negative at D28 compared with those who were parasitaemic.

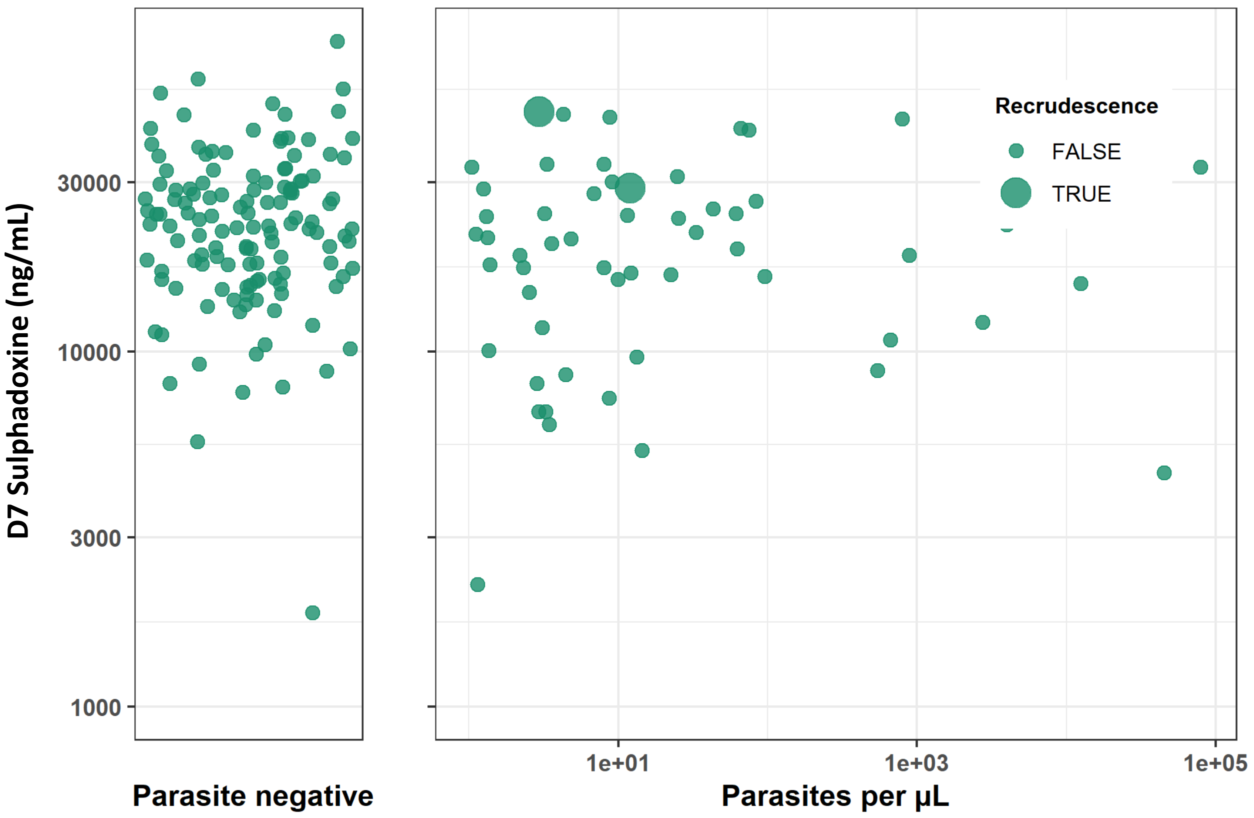

**Figure S7**. Day 7 capillary whole blood pyrimethamine concentrations in children whose blood samples were malaria parasite negative at D28 compared with those who were parasitaemic.

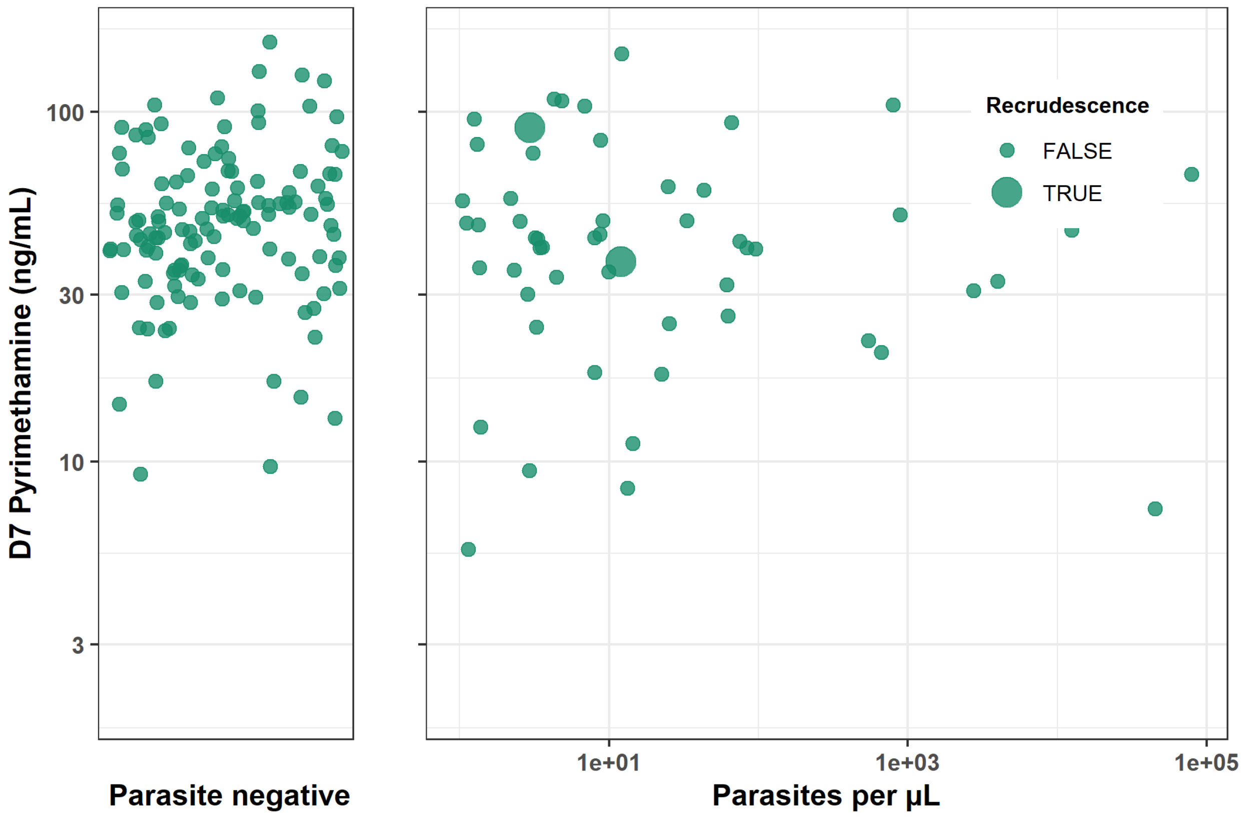

**Figure S8.** Proportions (95%CI) of children who were malaria parasitaemic on D28 in relation to the preceding day 7 sulphadoxine (left) and pyrimethamine (right) concentrations.

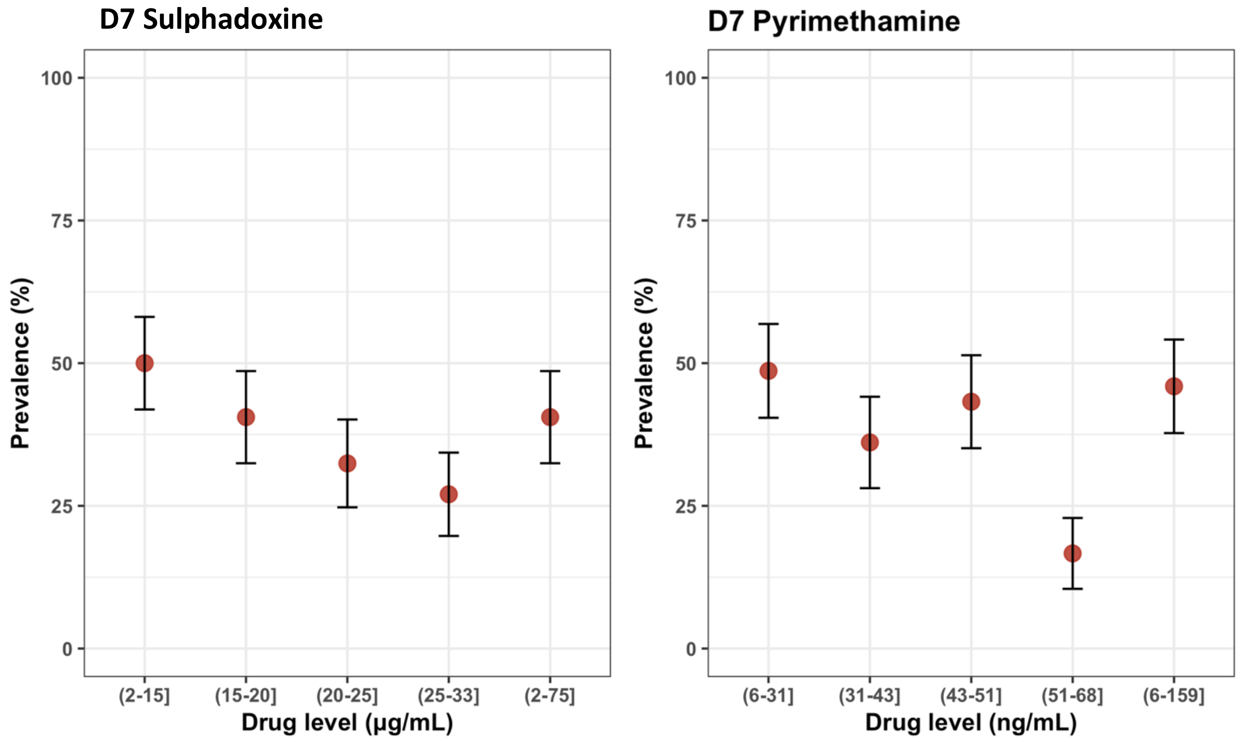

**Figure S9**: Relationship between malaria prevalence and parasite density across different weight bands. (a) malaria prevalence for each weight band, with error bars representing the 95% confidence intervals. (b) Parasite density in relation to weight, with data points colour-coded by age group

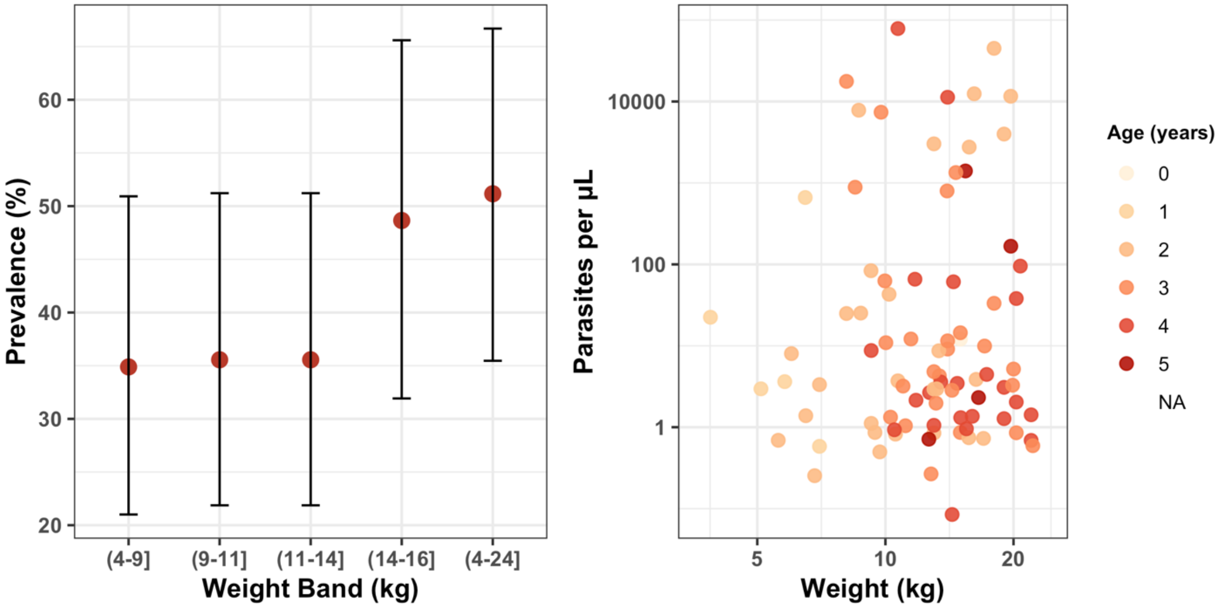

**Figure S10.** Predicted relationship between D28 desethylamodiaquine levels and breakthrough parasitaemia.

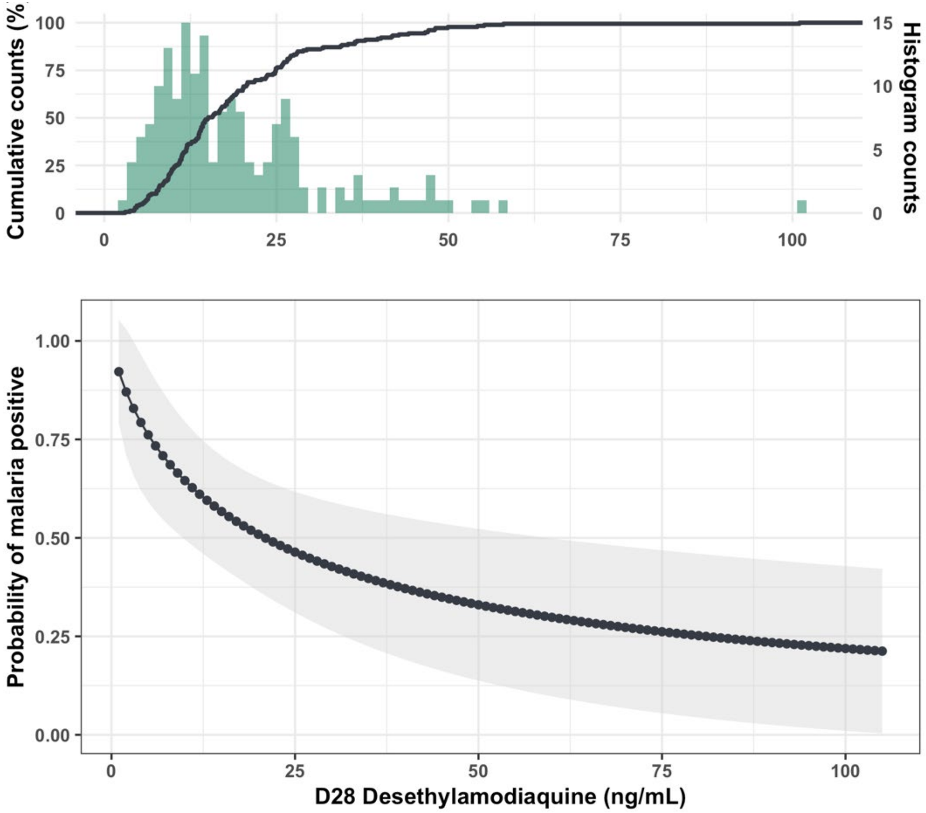

**Figure S11**: The relationship between the D7 whole blood desethyl amodiaquine concentrations and the area under the concentration-time curve (AUC$_{7-28}$) from D7 to D28

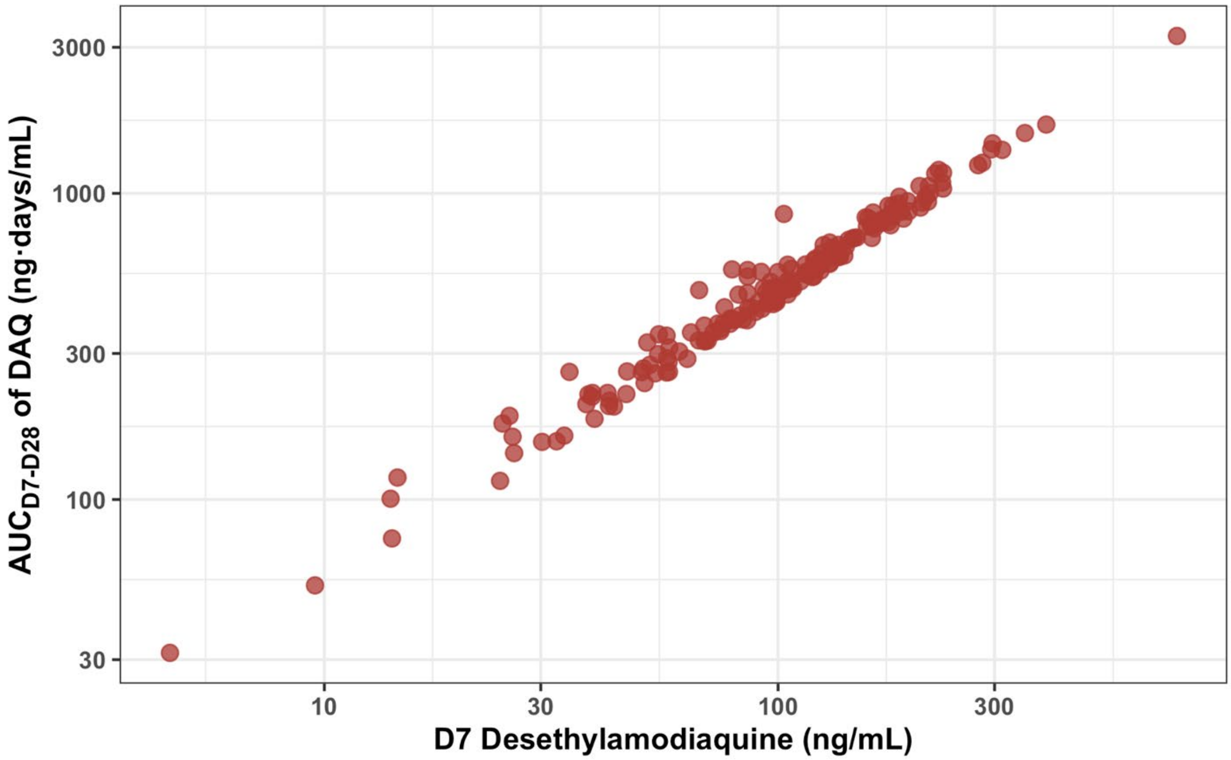

**Figure S12.** The relationship between between desethylamodiaquine and sulphadoxine estimated elimination half-lives

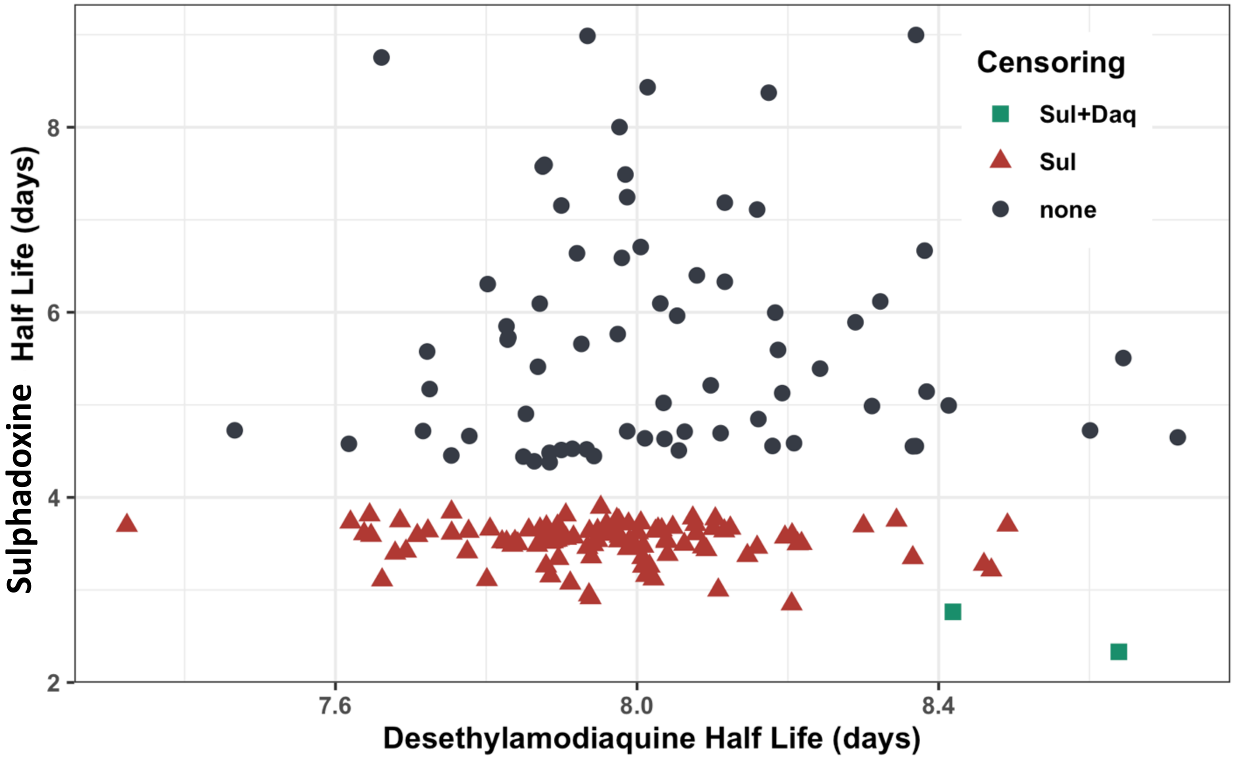

## Supplementary Tables

**Table S1**: The prevalence of malaria based on samples collected from children with data available for both dates.

| | Before SMC | After SMC |
|---|---|---|
| Malaria positive (PCR) | 139/194 (72%) | 88/194 (45%) |
| *P. falciparum* | 127/194 (66%) | 83/194 (43%) |
| *P. ovale* | 54/194 (28%) | 2/194 (1%) |
| *P. vivax* | 1/194 (0.5%) | 0/194 (0%) |
| *P. malariae* | 0/194 (0%) | 0/194 (0%) |
| Undetermined* | 4/194 (2%) | 2/194 (4%) |

Data are presented as n (%)

*If there was insufficient DNA then the malaria species could not be determined.

**Table S2.** Details of recrudescent *P. falciparum* infections.

| | Age (months) | Parasite density (per mL) at D28 | Desethylamodiaquine concentration (ng/mL) at D28 |
|---|---|---|---|
| 1 | 26 | 7,380,303 | 5.24 |
| 2 | 11 | 2,962 | 14.4 |
| 3 | 4 | 12,054 | 20.6 |

**Table S3: Summary of detectable drug levels**

| Drug name | Detectable Samples (n) | Geometric mean (ng/mL) | Minimum (ng/mL) | Maximum (ng/mL) |
|---|---|---|---|---|
| **Sulphadoxine** | | | | |
| D7 | 270/301 | 19402 | 1300 | 76100 |
| D28 | 102/248 | 2349 | 843 | 57100 |
| **Pyrimethamine** | | | | |
| D7 | 266/301 | 42 | 4.67 | 162 |
| D28* | 7/248 | 45 | 4.53 | 356 |
| **Amodiaquine** | | | | |
| D7 | 54/302 | 4 | 1.88 | 177 |
| D28* | 19/251 | 14 | 2.05 | 374 |
| **Desethylamodiaquine** | | | | |
| D7 | 274/302 | 74 | 3.19 | 757 |
| D28 | 243/251 | 8 | 3.01 | 357 |

* All detectable levels of sulphadoxine and amodiaquine were suspected to result from participants receiving their second round of SMC before, rather than after, the blood sample was taken.

Table S4: STROBE checklist

STROBE Statement—Checklist of items that should be included in reports of ***cohort studies***

| | Item No | Recommendation | Line No |
|---|---|---|---|
| **Title and abstract** | 1 | (*a*) Indicate the study's design with a commonly used term in the title or the abstract | 1-3 |
| | | (*b*) Provide in the abstract an informative and balanced summary of what was done and what was found | 33-56 |
| **Introduction** | | | |
| Background/rationale | 2 | Explain the scientific background and rationale for the investigation being reported | 61-75 |
| Objectives | 3 | State specific objectives, including any prespecified hypotheses | 75-78 |
| **Methods** | | | |
| Study design | 4 | Present key elements of study design early in the paper | 77-78, 84-85 |
| Setting | 5 | Describe the setting, locations, and relevant dates, including periods of recruitment, exposure, follow-up, and data collection | 82-84 86-98 101-117 |
| Participants | 6 | (*a*) Give the eligibility criteria, and the sources and methods of selection of participants. Describe methods of follow-up | 101-107 |
| | | (*b*) For matched studies, give matching criteria and number of exposed and unexposed | |
| Variables | 7 | Clearly define all outcomes, exposures, predictors, potential confounders, and effect modifiers. Give diagnostic criteria, if applicable | 122-134 |
| Data sources/ measurement | 8* | For each variable of interest, give sources of data and details of methods of assessment (measurement). Describe comparability of assessment methods if there is more than one group | 137-141 |
| Bias | 9 | Describe any efforts to address potential sources of bias | |
| Study size | 10 | Explain how the study size was arrived at | |
| Quantitative variables | 11 | Explain how quantitative variables were handled in the analyses. If applicable, describe which groupings were chosen and why | 136-141 |
| Statistical methods | 12 | (*a*) Describe all statistical methods, including those used to control for confounding | 136-141 |
| | | (*b*) Describe any methods used to examine subgroups and interactions | |

| | | | |
|---|---|---|---|
| | | (*c*) Explain how missing data were addressed<br>(*d*) If applicable, explain how loss to follow-up was addressed<br>(*e*) Describe any sensitivity analyses | 154-238 |
| **Results** | | | |
| Participants | 13* | (a) Report numbers of individuals at each stage of study—eg numbers potentially eligible, examined for eligibility, confirmed eligible, included in the study, completing follow-up, and analysed<br>(b) Give reasons for non-participation at each stage<br>(c) Consider use of a flow diagram | Fig 1 |
| Descriptive data | 14* | (a) Give characteristics of study participants (eg demographic, clinical, social) and information on exposures and potential confounders<br>(b) Indicate number of participants with missing data for each variable of interest<br>(c) Summarise follow-up time (eg, average and total amount) | Tables 144-167 |
| Outcome data | 15* | Report numbers of outcome events or summary measures over time | 169-265 |

| | | | |
|---|---|---|---|
| Main results | 16 | (*a*) Give unadjusted estimates and, if applicable, confounder-adjusted estimates and their precision (eg, 95% confidence interval). Make clear which confounders were adjusted for and why they were included<br>(*b*) Report category boundaries when continuous variables were categorized<br>(*c*) If relevant, consider translating estimates of relative risk into absolute risk for a meaningful time period | 169-265 |
| Other analyses | 17 | Report other analyses done—eg analyses of subgroups and interactions, and sensitivity analyses | supplement |
| **Discussion** | | | |
| Key results | 18 | Summarise key results with reference to study objectives | 268-356 |
| Limitations | 19 | Discuss limitations of the study, taking into account sources of potential bias or imprecision. Discuss both direction and magnitude of any potential bias | 3183-343 |
| Interpretation | 20 | Give a cautious overall interpretation of results considering objectives, limitations, multiplicity of analyses, results from similar studies, and other relevant evidence | 343-356 |
| Generalisability | 21 | Discuss the generalisability (external validity) of the study results | 353-356 |
| **Other information** | | | |
| Funding | 22 | Give the source of funding and the role of the funders for the present study and, if applicable, for the original study on which the present article is based | 380-385 |
